# Supplementary material for: The Use of Robotic Technology in the Healthcare of People above the Age of 65—A Systematic Review
Source: Healthcare (Basel). 2023 Mar 21;11(6):904. doi: 10.3390/healthcare11060904 (PMC10048377; doi:10.3390/healthcare11060904)
Supplement: Supplementary file 1 [file healthcare-11-00904-s001.zip › healthcare-2273704-supplementary.pdf]

Table S1. Characteristics of articles included in the systematic review of studies of the use of robotic technology in the healthcare of older people.

First author, Year, Country	Title	Aim	Robotic technology	Sample, including participants' characteristics
Chen et al.[a], 2020, Taiwan	A social robot intervention on depression, loneliness, and quality of life for Taiwanese older adults in long-term care	To investigate the effect of a social robot intervention on depression, loneliness, and quality of life of older adults in long-term care and to explore participants' experiences and perceptions after the intervention.	PARO* (Personal Assistive Robot) an animal companion robot. PARO appearance of a baby seal, equipped with tactile sensors that monitor sound, light, and touch. PARO can show human-like emotional reactions such as happiness and anger.	A purposive sample of 20 older adults. Age: 65-93 years (mean 81.1 years) Gender: 13 women and 7 men Diagnosis: Depression
Chen et al.[b,] 2020, Hong Kong	Changes in technology acceptance among older people with dementia: the role of social robot engagement	To investigate the technology acceptance among LTC (long term care) facilities' residents with dementia after direct exposure with a humanoid social robot (Kabochan).	Kabochan, a smart, social, humanoid robot with a warm texture, which is 22× 18 × 28 cm, weighs 970 g, and is made to resemble a three-year-old boy. Kabochan has sensors installed in the mouth, head, hands, feet, and the main body, which allow it to engage through talking, singing, and head nodding as a response to its users' various audio and motor stimulations. Pre-programmed with 400 conversational phrases in Japanese to promote communication.	A sample of 103 residents. Age: 67-108 years (mean 87.2 years) Gender: 82 women and 21 men Diagnosis: Dementia 76.7% severe dementia and 23.3% moderate dementia

Chu et al., 2017, Australia	Service innovation through social robot engagement to improve dementia care quality	To show how the engagement between two social robots and persons with dementia in Australian residential care facilities can improve care quality.	Sophie and Jack: social babyface robots designed specifically for emotional and intentional communication and interaction purposes. Upper part contains two light sensors, two microphones for speech recognition, and voice localization in the front. It's head has two axes of movement, nodding/ panning ± 180° and tilting ± 45°, 39 cm tall and weighs 6.5 kg. Enable face recognition, subject registration and tracking, emotion change recognition, voice vocalization, gestures, emotive expressions, singing, and dancing.	A sample of 139 residents.<br>Age: 65-90 years<br>Gender: 95 women and 44 men<br>Diagnosis: Different levels of dementia
Fan et al., 2021, United States	Field testing of Ro-Tri, a robot-mediated triadic interaction for older adults	To explore the feasibility, acceptability, and effect of the Ro-Tri socially assistive robotic (SAR) system on engagement and social interaction of older adults residing in long term care settings and whether Ro-Tri	Ro-Tri- a socially assistive robotic system (with NAO and the Kinect). Administrating four activities: Simon Says, Book sorting - both simultaneous and take turn, and Book sorting with additional rules.	A sample of 7 pairs of older adults<br>Age: mean 82,7 years<br>Diagnosis:<br>Normal cognition: 3 persons<br>Mild cognitive impairment: 10 persons<br>Alzheimer's dementia: 1 person

		SAR was capable of delivering multimodal stimuli involving physical, cognitive and social stimuli to older adults in such settings over a longer duration.		
Fields et al., 2021, United States	Shall I compare thee … to a robot? An exploratory pilot study using participatory arts and social robotics to improve psychological well-being in later life	To improve the psychological well-being of study participants both with and without cognitive impairment who live in a residential care setting.	NAO- a theatre and social robot performing and concurrently encouraging the older adult to perform (specifically Shakespeare's text), as a function of a participatory performance arts model.	A purposive sample of 15 older adults<br>Age: 77-92 years (mean 85.8 years)<br>Gender: 11 women and 4 men<br>Diagnosis: No cognitive impairment: 8; Mild cognitive impairment: 5; Moderate cognitive impairment: 2
Gustafsson et al., 2016, Sweden	Using a robotic cat in dementia care.<br>A pilot study	To explore the reactions of individuals with dementia to an interactive robotic cat and their relatives' and professional caregivers' experiences regarding its usability, function, and effects.	JustoCat®- an interactive robotic pet developed using reminiscence therapy. Easy-to-change fur facilitating personalized use, washable fur following hygiene routines required in nursing homes and hospital settings in Sweden.	A purposive sample of four individuals.<br>Age: 82-90 years<br>Gender: 2 women and 2 men<br>Diagnosis: late stage of dementia<br>Three relatives participated in the interview<br>(wife or son).<br>11 professional caregivers, including 2 RNs, 1 occupational therapist, and 8 assistant nurses.
Khosla et al., 2021, Australia	Engagement and experience of older people with socially assistive robots in home care	To study the engagement and robot experience of older people with dementia	Betty- a social robot. 39 cm tall and weighs 6.5 kg. The upper part contains two light sensors, two microphones for speech	Five older people who live with at least one family member<br>Age: 75-85 years<br>Diagnosis: dementia with multiple medical conditions

		while interacting with a social robot named Betty in the context of home-based care.	recognition, and voice localization in the front. It's head has two axes of movement, nodding/ panning ± 180 degrees and tilting ± 45 degrees. Involves modelling of human characteristics like gesture, emotional expressions, voice, and motion. Betty is personalized to each participant based on the direct consultations and face-to-face meetings	
Papadopoulos et al., 2021, United Kingdom	The CARESSES Randomised Controlled Trial: Exploring the Health-Related Impact of Culturally Competent Artificial Intelligence Embedded into Socially Assistive Robots and Tested in Older Adult Care Homes	To develop and evaluate a culturally competent artificial intelligent system embedded into social robots to support older adult wellbeing.	Pepper- a culturally competent socially assistive robot used to supplement existing older adult care in long-term residential care settings.	A sample of 33 residents. Age: 65-98 years (mean 81.9 years) Gender: 22 women and 11 men Diagnosis: None stated
Pu et al., 2020, Australia	How people with dementia perceive a therapeutic robot called PARO in relation to their pain and mood: A qualitative study	To explore how people with mild to moderate dementia and chronic pain perceive PARO as an alternative intervention to manage their pain and mood.	PARO*	A sample of 11 participants Age: 65-94 years (mean 84.36 years) Gender: 81.82% women Diagnosis: dementia, mild to severe pain

Robinson et al., 2013, New Zealand	Suitability of healthcare robots for a dementia unit and suggested improvements	To investigate the suitability of a new eldercare robot (Guide) for people with dementia and their caregivers compared with one that has been successfully used before (PARO), and to generate suggestions for improved robot enhanced dementia care.	Guide- a 1.6 meter tall robot, with a head and a large touch screen for interaction. The robot interacts with the user by speaking, displaying messages/ images/video/text on a touch screen and accepting user input on the touch screen. Can be programmed with software applications, which currently include the ability to take vital signs and store them in a database, entertainment (music videos, quotes, photographs), telephone calling to phone numbers using Skype, and brain fitness games.<br>PARO*	A sample of 10 people<br>Age: 71-93 years<br>Gender: 5 women and 5 men<br>Diagnosis: dementia<br>11 of their family members (age: 42-88 years, gender: 4 women and 7 man)<br>5 staff members (age: 45-66 years, gender: 5 women, length of employment 4 months to 14 years)
Robinson et al., 2015, New Zealand	Physiological effects of a companion robot on blood pressure of older people in residential care facility: A pilot study	To investigate the effects of interacting with the companion robot, PARO, on blood pressure and heart rate of older people in a residential care facility.	PARO*	A sample of 21 residents: 10 from the hospital and 11 from the rest home.<br>Age: 71 – 95 years (mean 84.9 years)<br>Gender: 14 women and 7 men
Robinson et al., 2016, New Zealand	Group sessions with PARO in a nursing home:	To describe group sessions and through qualitative	PARO*	A sample of 20 residents from the PARO group (7 from the rest home and 13 from the hospital)

	Structure, observations and interviews	study, gain insight into the experiences of people interacting with the robot and how such interactions could reduce loneliness.		Age: mean 84.40 years<br>Gender: 14 women and 6 men<br>Diagnosis: Cognitive impairment (8 participants) Poor or fair health (7 participants) Good or excellent health (13 residents)<br>A sample of 21 staff<br>Age: mean 47.04 years<br>Gender: 21 women
Sumioka et al., 2021, Japan	A minimal design of a human infant presence: A case study toward interactive doll therapy for older adults with dementia	To experiment-ally investigate how HIRO and a baby robot with a face influence older adults in their living environments<br>To evaluate whether seniors with dementia engage in positive, 5- minutes interactions with HIRO or a baby robot with a face.<br>To observe the interaction between older people and the robots, and identify their common and unique responses.	HIRO, which shape and size resemble a human baby, but with eliminated facial and emotional expressions as well as detailed body parts such as hands. (W210xD165xH300mm and 610g). The recorded voice of an actual human infant with multiple emotional states is applied to strengthen a feeling of human infant presence and facilitate emotional interactions. HIRO vocalizes based on its' emotional states, which respond to the actions of older adults as well as its own internal mechanism	A sample of 21 older participants.<br>Age: mean 86.6 years (SD 5.4).<br>Gender: 18 women and 3 men<br>Diagnosis: Dementia
Werner et al., 2018, Germany	User-oriented evaluation of a robotic rollator that provides navigation	To determine whether the robotic rollator	MOBOT- an Intelligent Active Mobility Assistance Robot.	A sample of 42 frail older persons.

	assistance in frail older adults with and without cognitive impairment	(RR) provided navigation assistance improves navigation within a real-life environment in the intended user group of frail older adults with and without cognitive impairment currently using a rollator in daily life.	The MOBOT rollator-type mobility assistant integrates innovative functionalities such as obstacle detection and avoidance, indoor localization and navigation assistance, user following, gait tracking, and audio-gestural human-robot interaction into an overall context-aware mobility assistance robot.	Age: mean 82.5 years<br><br>Diagnosis: Mild to moderate cognitive impairment (20 persons)<br>Without cognitive impairment (21 persons)
